# Long-Term Efficacy of Safinamide on Symptoms Severity and Quality of Life in Fluctuating Parkinson’s Disease Patients

**DOI:** 10.3233/JPD-191765

**Published:** 2020-01-13

**Authors:** Carlo Cattaneo, Wolfgang H. Jost, Erminio Bonizzoni

**Affiliations:** a Medical Department, Zambon SpA, Bresso, Italy; b Parkinson-Klinik Ortenau, Wolfach, Germany; c Department of Clinical Science and Community, Section of Medical Statistics and Biometry “GA Maccacaro”, University of Milan, Milan, Italy

**Keywords:** Parkinson’s disease, safinamide, MAO-B inhibitor, quality of life, motor fluctuations, glutamate, adjunct therapy

## Abstract

**Background:**

Parkinson’s disease (PD) is characterized by a wide range of motor and non-motor symptoms. Levodopa is still the most effective drug; however, its long-term use is associated with motor complications which may deteriorate patient’s quality of life. Safinamide is a unique treatment modulating both dopaminergic and glutamatergic systems. Previous results from two six months, double-blind, placebo-controlled studies demonstrated that safinamide has positive effects on both motor functions and quality of life in PD patients.

**Objective:**

To investigate the effects of safinamide 100 mg/day over two-year treatment on PD symptoms severity and quality of life, using data from the study 018.

**Methods:**

Data from 352 patients were analyzed to evaluate the effects of safinamide on OFF time and ON time (with no or non-troublesome dyskinesia) in the overall population and in subgroups of patients (receiving levodopa monotherapy or with other anti-Parkinson therapies), and the effects of safinamide on motor symptoms/clinical fluctuations (by means of UPDRS III and IV) and on health-related quality of life (using UPDRS II and PDQ-39 summary index score).

**Results:**

Safinamide, administered as add-on to standard therapy in fluctuating PD patients, significantly improved motor symptoms and clinical fluctuations in the overall population and in some subgroups of patients. Additionally, safinamide improved quality of life and activities of daily living, maintaining the efficacy in the long-term.

**Conclusions:**

The findings of these analyses suggest that safinamide may be considered an appropriate adjunct therapy in patient not sufficiently controlled. Further investigations are desirable to confirm these results in usual care setting.

## INTRODUCTION

Parkinson’s disease (PD) is the second most common neurodegenerative brain disorder of the elderly, characterized by a progressive functional loss of nigrostriatal dopaminergic neurons leading to a classical set of motor symptoms (resting tremor, rigidity, bradykinesia and postural instability) [1]. Several non-motor symptoms (such as neuropsychiatric and sleep disturbances, pain, autonomic dysfunctions) frequently develop prior to the occurrence of motor impairment with a negative impact on patients’ quality of life [2]. Improvement of motor symptoms can also improve non-motor symptoms and this heterogeneity of clinical symptoms requires an individual tailored treatment [3].

The incidence of PD increases with age rising from 0.3 per 1,000 person-years in patients aged 55 to 65 years, to 4.4 per 1,000 person-years for those aged ≥85 years [4]. The medical expenditure of PD is one of the highest ranked of the neurological diseases, which could be the main cause of a serious socioeconomic burden in the future aging society [5].

The understanding that PD is a syndrome of dopamine deficiency led to the introduction into clinical practice of levodopa (L-dopa), a precursor of dopamine that crosses the blood brain barrier.

L-dopa remains the gold standard therapy for PD. However, as the disease progresses, it becomes less controllable and motor complications, including fluctuations and levodopa-induced dyskinesia (LID), eventually develop [6].

Nearly 40% of PD patients develop motor fluctuations after 4 to 6 years of L-dopa treatment, and up to 90% develop LIDs after 10 years, depending on several factors, such as age, duration and dose of L-Dopa medication [7].

Increasing evidence is showing that in addition to neurodegeneration of the nigrostriatal dopaminergic neurons, dysregulation of other neurotransmitter systems in different brain areas, particularly of glutamate, are also implicated in the pathophysiology of the disease and the complications [8]. Targeting non-dopaminergic systems is thus a complementary approach to improve and control such motor complications, while maintaining the efficacy of L-dopa [9].

Safinamide is a multimodal drug, with a dual mechanism of action that includes reversible monoamine-oxidase B (MAO-B) inhibition and glutamate modulation. This broad spectrum of action corresponds to the multiple neurochemical alterations in PD and may provide a comprehensive symptomatic relief for the patients [10]. Biogenic amines (such as dopamine) and the inhibitors concentrations influence the peripheral monoamine oxidase enzyme activity in chronic levodopa-treated patients [11]. Safinamide, due to its high selectivity for MAO-B and its reversibility, does not affect MAO-A activity and does not require a specific diet restriction [12].

Safinamide has been shown to reduce OFF time, increase ON time and improve some non-motor symptoms such as pain, sleep disorders and mood deteriorations, probably due to its dual dopaminergic and glutamatergic mechanism of action [13–16].

A previous analysis of the pooled data from two six months pivotal trials (studies 016 and SETTLE) provided clinically relevant information about the effect of adjunctive safinamide treatment, at the dose of 100 mg/day, on motor symptoms and motor complications in specific patient subgroups [17–19].

The aim of this new analysis of the data from the two years study 018 is to evaluate the long-term clinical effects of safinamide 100 mg/day, administered as add-on therapy to L-dopa in fluctuating PD patients, on symptoms severity and quality of life.

## MATERIALS AND METHODS

### Study design

Trial 018 (NCT01286935) was a multicenter, multinational, double-blind, placebo-controlled, extension of the Phase III study 016 in fluctuating PD patients. Subjects were included in study 018 if they had completed trial 016, were treatment compliant and willing to continue. After finishing the initial treatment period, patients entering the extension phase continued on their randomized study medications and were followed-up up to two years [20].

Safinamide or placebo were given as add-on to L-dopa and other PD therapies (dopamine agonists, catechol-O-methyltransferase inhibitors, amantadine). The doses of L-dopa and other PD treatments were optimized before the study start and should remain stable, whenever possible, during the trial. However, in case of motor symptom deterioration, dose increases of L-dopa or additional PD drugs, except safinamide, were permitted. The L-dopa dose could also be decreased based on the occurrence of adverse events.

Treatments with tri-tetracyclics, MAO-inhibitors, serotonin-norepinephrine reuptake inhibitors (SNRIs), opioids, neuroleptics, barbiturates and phenothiazines were not permitted. Selective serotonin reuptake inhibitors (SSRIs) were allowed at study entry at the lowest therapeutic dose and had to remain stable throughout the trial.

Both the protocol and patient materials were approved by Independent Ethics Committees and Health Authorities in all the participating countries. All patients signed an informed consent form and the study was conducted according to the ethical standards of the institutional and/or national research committee and to the Declaration of Helsinki.

The primary endpoint of study 018 was the change from baseline over two years in the Dyskinesia Rating Scale (DRS) total score and the main secondary endpoints were the change in the total daily ON and OFF time. All the scales/questionnaires administered during the study were performed during the ON phase. The significant improvements in ON and OFF time seen at six months were maintained up to two years. Although there was no overall difference in dyskinesia, despite a substantial decrease in DRS scores with safinamide 100 mg/day compared with placebo (27% vs 3% respectively), a significant improvement (*p* = 0.0317) was seen in patients with DRS >4 at baseline.

The incidence of adverse events (AEs) and serious adverse events was similar in safinamide and placebo groups, and most AEs were rated as mild or moderate. No significant abnormalities were detected in cardiovascular, laboratory or ophthalmological examinations between treatment groups [20].

### Outcome measures

This is a *post hoc* analysis of the data of study 018, comparing the effects of safinamide 100 mg once daily versus placebo on symptoms severity (motor symptoms and clinical fluctuations) and quality of life.

*Motor symptoms* were analyzed by means of the changes from baseline (study 016 start) to two years in the Unified Parkinson’s Disease Rating Scale (UPDRS) part III scores during the ON time. The UPDRS [21] is the most commonly used scale in clinical studies to follow the longitudinal clinical course of PD and is rated by the Investigator. It comprises four parts: part III is used to evaluate motor functions and contains 27 items, with each item scored on a 5-point scale (from 0 to 4). The total score of part III may range from 0 (no disability) to 108 (total disability).

*Motor fluctuations* were evaluated using:

- the changes from baseline to two years in the scores of the items 36–39 of UPDRS part IV (complications of therapy) during ON time. Items 36–38 are scored from 0 to 1, item 39 from 0 to 4. The total score may thus range from 0 to 7.
- the mean change from baseline to two years in daily OFF time, as recorded by patients in home diaries [22].
- the mean change from baseline to two years in daily ON time with no or non-troublesome dyskinesia as measured by patients’ diary cards. Non-troublesome dyskinesia was defined as dyskinesia that did not interfere with function or cause significant discomfort.

ON and OFF time were also evaluated in subgroups of patients receiving only L-dopa at baseline (i.e., no concomitant treatment with other anti-Parkinson drugs), those considered (or not) “mild fluctuators” at baseline (daily OFF time ≤4 h irrespective of concomitant medication) and those who were or not receiving a concomitant dopamine agonist (DA), or a concomitant catechol-O-methyltransferase (COMT) inhibitor or concomitant amantadine.

*Health-related quality of life* were measured using the UPDRS part II scale and the Parkinson’s Disease Questionnaire-39 items (PDQ-39) summary index score.

- The UPDRS part II evaluates activities of daily living and contains 13 items, with each item scored on a 5-point scale (from 0 to 4). The total score of part II may range from 0 (no disability) to 52 (total disability).
- The PDQ-39 (23) is a disease-specific, patient-reported outcome and comprises 39 questions measuring eight dimensions of health: mobility, activities of daily living, emotional well-being, stigma, social support, cognition, communication and bodily pain. Each of the 39 items is rated using a 5-point Likert scale, with 0 for never having difficulties/problems and 4 for always having difficulties/problems. The total score ranges from 0 to 156 with higher scores indicating worse health status. The PDQ-39 summary index score is obtained by summing all scores and standardizing (dividing per eight) this sum on a 0–100 scale.

### Statistical methods

For all the efficacy endpoints the basal value was the value at the start of study 016. Comparisons of the mean change from baseline to two years for the active-treatment group to placebo were performed using linear effects models with treatment group and study index as fixed dummy effects and baseline value as continuous covariate (ANCOVA analyses). Results are reported as least square means with associated standard errors, two-tailed 95% confidence intervals (CIs) and two-tailed *P*-values. The intention-to-treat (ITT) patient populations were used for all *post hoc* analyses while the last observation carried forward (LOCF) approach was applied to account for missing data at study termination. No *P*-value adjustments were made for multiplicity generated by secondary and subgroup analyses. SAS software version 9.4 was used for all statistical analyses.

## RESULTS

A previous *post-hoc* analysis on the data after six months of treatment showed a significant improvement in motor symptoms and motor fluctuations [17].

This report expands on the previous study by long-term data up to two years.

### Motor symptoms

Safinamide 100 mg significantly improved mean UPDRS Part III (motor symptoms) scores during ON time by –6.06 points (95% CI: –6.85, –5.00) from baseline, compared with –3.94 points (95% CI: –4.40, –2.68) with placebo (*p* = 0.0063; mean difference between safinamide and placebo –2.13 points, see Table 1).

**Table 1:** Study 018: changes from baseline and differences of changes in UPDRS scores and PDQ-39 summary index score

Outcome	Change with safinamide 100 mg (*n* = 178) mean [95% CI]	Change with placebo (*n* = 174) mean [95% CI]	Difference safinamide vs placebo mean [95% CI]	*P* value^*^
UPDRS II (ADL)	–1.97 [–2.11, –1.40]	–0.91 [–1.11, –0.28]	–1.06 (–1.83, –0.29)	0.0068
UPDRS III (Motor symptoms)	–6.06 [–6.85, –5.00]	–3.94 [–4.40, –3.00]	–2.13 (–3.65, –0.60)	0.0063
UPDRS IV (Clinical Fluctuations)	–0.54 [–0.66, – –0.41]	–0.27 [–0.40, –0.14]	–0.27 (–0.44, –0.09)	0.0035
PDQ-39 SI score	–4.07 [–5.68, –2.45]	–1.63 [–3.29, 0.03]	–2.44 [–4.75, –0.12]	0.0390

Mean values are least squares estimates. N, number of patients; CI, confidence interval; ADL, activities of daily living; UPDRS, Unified Parkinson’s Disease Rating Scale; SI, summary index. ^*^p value versus placebo calculated using ANCOVA models with treatment and study as fixed effects and baseline value as continuous covariate.

### Motor fluctuations

#### UPDRS IV – items 36–39

There was a statistically significant difference between safinamide and placebo cohorts regarding the scores of the items 36–39 (clinical fluctuations) of the UPDRS Part IV scale: –0.54 points (95% CI: –0.66, –0.41) from baseline with safinamide 100 mg, compared with –0.27 points (95% CI: –0.40, –0.14) with placebo (*p* = 0.0035; mean difference between safinamide and placebo –0.27 points, see Table 1).

#### Daily OFF time and daily ON time with no/non-troublesome dyskinesia

The intention-to-treat (ITT) population (352 subjects in total) included the patients who entered into the study 018. When added to optimized dopaminergic treatment, safinamide 100 mg significantly reduced the mean total daily OFF time by –1.67 hour (95% CI: –1.98, –1.36) from baseline, compared with a reduction of –1.00 hour (95% CI: –1.31, –0.68) observed with placebo (*p* < 0.0031; mean difference between safinamide and placebo – 0.67 hour). Similarly, safinamide significantly improved, compared with placebo, the daily ON time with no or non-troublesome dyskinesia: 1.60 hour (95% CI: 1.25, 1.95) versus 0.90 hour (95% CI: 0.54, 1.26; *p* < 0.0067; mean difference between safinamide and placebo 0.70 hour).

**Fig.1:**
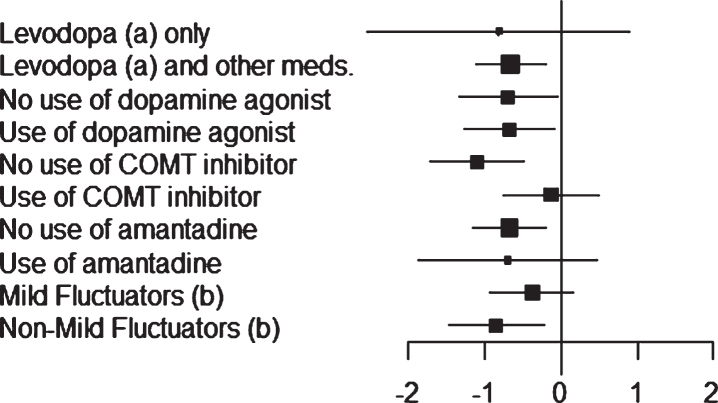
Study 018: forest plot (OFF time) of the subgroups. Mean difference between safinamide and placebo with 95% Confidence Interval (CI).

**Fig.2:**
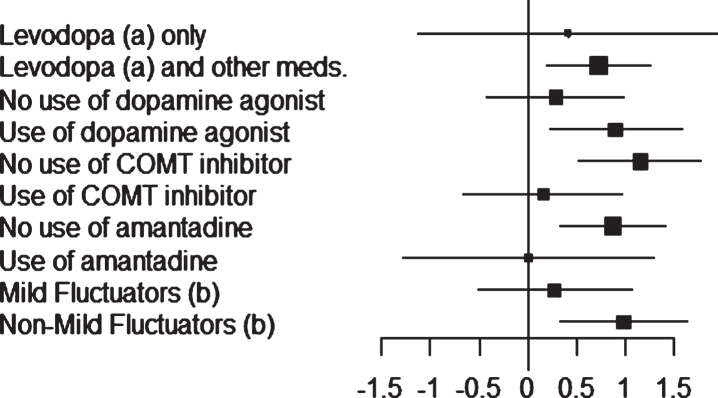
Study 018: forest plot (ON time with no/non-troublesome dyskinesia) of the subgroups. Mean difference between safinamide and placebo with 95% Confidence Interval (CI).

Least-squares estimates of changes in OFF time and ON time (with no or non-troublesome dyskinesia) obtained for the different patient stratifications are shown in Figs. 1 and 2.

About 93% of patients (165 with safinamide, 161 with placebo) were treated with L-dopa and other dopaminergic medications. Regarding the OFF time, a significant reduction by – 1.68 hour (95% CI: –2.01, –1.36) was seen with safinamide compared to –1.02 hour with placebo (95% CI: –1.35, –0.69; *p* < 0.0051; mean difference between safinamide and placebo –0.66 hour). The same result was obtained for the ON time (with no or non-troublesome dyskinesia): safinamide increased the mean by 1.61 hour (95% CI: 1.23, 1.98), significantly more than placebo: 0.88 hour (95% CI: 0.50, 1.25; *p* = 0.0073; mean difference between safinamide and placebo 0.73 hour).

Only 7% of patients (13 per group) were treated with L-dopa alone. Despite a difference of –0.81 hour for the OFF time and 0.41 hour for the ON time with no/non-troublesome dyskinesia in favor of safinamide, the results were not statistically significant (*p* = 0.3625 for the OFF time and *p* = 0.6062 for the ON time), probably due to the low number of patients.

### Concomitant dopamine agonist (DA) use

In study 018, about 64% of patients (110 with safinamide, 116 with placebo) were taking stable doses of a DA in addition to L-dopa at randomization.

For the patients already on DA, adding safinamide 100 mg significantly reduced the mean daily OFF time by –1.71 hour (95% CI: –2.14, –1.29), compared to –1.03 hour with placebo (95% CI: –1.45, –0.62; *p* = 0.0259; mean difference between safinamide and placebo –0.68 hour). The same result was observed for the patients not taking baseline DA medications: –1.60 hour with safinamide (95% CI: –2.04, –1.16), as opposed to –0.91 hour with placebo (95% CI: –1.39, –0.43; *p* = 0.0368; mean difference between safinamide and placebo –0.69 hour).

Concomitantly, adding safinamide in patients already on DA increased the mean daily ON time with no/non-troublesome dyskinesia (1.77 hour; 95% CI: 1.28, 2.25) significantly more than did placebo (0.87 hour; 95% CI: 0.40, 1.34; *p* < 0.0098; mean difference between safinamide and placebo 0.90 hour).

A lower mean effect size, not statistically significant (*p* = 0.436), was observed in the patients who were not taking DA as baseline medication: 1.29 hour for safinamide (95% CI: 0.81, 1.77), compared to 1.01 hour with placebo (95% CI: 0.49, –1.53; mean difference between safinamide and placebo 0.28 hour).

### Concomitant catechol-O-methyltransferase (COMT) inhibitor use

In the overall population, about 43% of patients (79 with safinamide, 74 with placebo) were taking stable doses of a COMT inhibitor in addition to L-dopa at randomization. In this subgroup of patients, the difference between safinamide and placebo was non-significant for both OFF time (–0.13 hour, *p* = 0.6841) and ON time with no/non-troublesome dyskinesia (0.15 hour, *p* = 0.7132).

On the contrary, patients not using COMT inhibitors at baseline showed a statistically significant reduction in the mean daily OFF time with safinamide (–1.84 hour; 95% CI: –2.28, –1.40) compared with placebo (–0.73 hour; 95% CI: –1.17, –0.30, *p* = 0.0005; mean difference between safinamide and placebo –1.10 hour).

The same result was obtained for the mean daily ON time with no/non-troublesome dyskinesia: significant increase by 1.94 hour with safinamide (95% CI: 1.49, 2.38) compared to 0.79 hour with placebo (95% CI: 0.35, 1.23; *p* = 0.0004; mean difference between safinamide and placebo 1.15 hour).

### Concomitant amantadine use

Only 19% of patients (33 with safinamide, 36 with placebo) were taking stable doses of amantadine in addition to L-dopa at randomization. Probably due to this low number of subjects, no difference was detected for the ON time with no/non-troublesome dyskinesia between safinamide and placebo, and only a slight difference, non-significant, for the OFF time (–0.70 hour, *p* = 0.2496).

On the contrary, the mean daily OFF time decreased with safinamide significantly more than with placebo in the patients not using amantadine: –1.61 hour (95% CI: –1.94, –1.28), as opposed to –0.94 hour with placebo (95% CI: –1.28, –0.60, *p* = 0.0057; mean difference between safinamide and placebo –0.67 hour), and the same effect was observed for the mean daily ON time with no/non-troublesome dyskinesia: increase by 1.59 hour with safinamide (95% CI: 1.21, 1.96) and 0.71 hour with placebo (95% CI: 0.32, 1.10, *p* = 0.0018; mean difference between safinamide and placebo 0.87 hour).

### Mild fluctuators subgroup

Mild fluctuators (patients experiencing ≤4h of daily OFF time) were about 35% of the overall ITT population (69 with safinamide, 60 with placebo). Despite a difference of –0.38 hour for the OFF time and 0.27 hour for the ON time with no/non-troublesome dyskinesia in favor of safinamide, the results were not statistically significant (*p* = 0.1802 for the OFF time and *p* = 0.4992 for the ON time).

The addition of safinamide to L-dopa in non-mild fluctuators significantly decreased the mean daily OFF time: –2.30 hour (95% CI: –2.71, –1.86), compared to –1.45 hour with placebo (95% CI: –1.89, –1.02; *p* = 0.0078; mean difference between safinamide and placebo –0.85 hour). Consistently, the mean daily ON time with no/non-troublesome dyskinesia significantly increased by 2.02 hour with safinamide (95% CI: 1.55, 2.48), as opposed to 1.04 hour with placebo (95% CI: 0.58, 1.49, *p* = 0.0035; mean difference between safinamide and placebo 0.98 hour).

### Health-related quality of life

Safinamide significantly improved mean UPDRS Part II (activities of daily living) scores during ON time by –1.97 points from baseline (95% CI: –2.11, –1.40), compared with –0.91 points with placebo (95% CI: –1.11, –0.28; *p* = 0.0068; mean difference between safinamide and placebo –1.06 points, see Table 1).

Further evidence of the benefit of safinamide on quality of life was provided by the PDQ-39 scale performed by the patients.

The mean decrease (improvement) in the PDQ-39 summary index score was –4.07 points (95% CI: –5.68, –2.45) with safinamide vs –1.63 points (95% CI: –3.29, 0.03) with placebo, with a statistically significant difference of –2.44 point (*p* = 0.0390, see Table 1).

## DISCUSSION

This *post-hoc* analysis showed that the clinical benefits observed after six months with safinamide across a range of different patient populations were mostly maintained in the long-term. The data were obtained through standardized scales (UPDRS part II, III and IV) scored by the Investigators and by patient-reported outcome measures (home-diary record of ON and OFF, PDQ-39 questionnaire).

The majority of patients (93%) were receiving L-dopa with one or more concomitant anti-Parkinson drugs. The severity of motor fluctuations increases with the duration of the L-dopa use [24]. Costs of both medical services and patient care increase with the progression of the disease, with a specific and significant relationship between the costs and the time spent in OFF state [25]. The results of the ITT population showed that adding safinamide to an optimized dopaminergic treatment regime can significantly reduce the mean total daily OFF time and significantly increase the ON time with no/non-troublesome dyskinesia. The latter is the so called “good” ON time and correlates with patients’ perceived duration of a good response to the therapy throughout the day [20].

These positive outcomes were confirmed by the significant improvements in the items of the UPDRS IV measuring the complications of the therapy.

Patients with motor fluctuations often require add-on therapies that can exacerbate dyskinesia, one of the most disabling side effects with a significant impact on patients’ quality of life. Rasagiline, in example, significantly increased the ON time with troublesome dyskinesia in the PRESTO study [26]. On the contrary, safinamide improved the motor symptoms without increasing the troublesome dyskinesia [27].

Similar efficacy was seen in patients treated with dopamine-agonists (DA), in those not treated with amantadine or not receiving concomitant COMT inhibitors (suggesting that safinamide could be a valid alternative to these treatments) and in the non-mild fluctuators. A trend in favor of safinamide was observed in the other subgroups of patients, although not statistically significant, with a minimal beneficial effect of the drug. In some cases (i.e., L-dopa monotherapy and concomitant amantadine use) this was probably due to the very low number of subjects not adequate for an appropriate powered statistical analysis.

Anyway, the combined treatment of amantadine and safinamide was very well tolerated, with no particular safety problems and an additional improvement of the OFF time.

Safinamide reduces presynaptic abnormal glutamate release, modulating the glutamatergic stimulation with consequent stabilization of mood and pain relief.

Amantadine also modulate glutamatergic stimulation by NMDA receptor antagonism, improving vigilance and dyskinesia. Thus, the combination of the two drugs may help PD patients to better tolerate the motor and non-motor features of the OFF time periods that characterize the long-term dopamine replacement therapy [28].

The improvements observed in the DA subgroups are particularly important. Two third of the patients in polytherapy with L-dopa in the study 018 were receiving a DA as add-on treatment, nevertheless they were experiencing motor fluctuations. In this case, increasing the DA dose to improve the symptoms can cause intolerable adverse effects, including somnolence, hallucinations and impulse control disorders. Adding safinamide when the therapeutic effect of the DA become suboptimal can improve the treatment efficacy without tolerability problems and with no need to increase the DA dose or switch to another drug.

Consistent with the benefits observed in motor fluctuations, there was a statistically significant improvement in the UPDRS scores for motor function in the overall population. The change from baseline in the UPDRS-III with safinamide represented a moderate clinically important difference (CID), according to criteria developed by Shulman et al.

A CID is the amount of change on a measure that patients recognize as clinically significant and valuable [29] and is one of the most important tools for patient-centered trials [30].

All these positive outcomes resulted in an improvement in the activities of daily living, as measured by the UPDRS part II scale, and in the patient’s quality of life. While in the past the clinical studies on PD were concentrated mainly on the assessment of motor functions, nowadays the focus includes also the evaluation of the impact of the disease on patients’ daily lives, their physical and psychological well-being and social participation. The magnitude of safinamide effect measured by the UPDRS II was equivalent to the minimal CID of pramipexole extended release in the same patient population [31].

These beneficial effects are also reflected by the statistically significant improvement in the PDQ-39 summary index score.

The PDQ-39 is the most thoroughly used health-related quality of life (HRQoL) questionnaire for PD, is one of the few scales considered disease specific and is a patient-reported outcome (PRO). The European Medicines Agency and the US Food and Drug Administration recommended the inclusion of PROs in clinical trials because, in contrast to the scales rated by clinicians, PROs are self-reported by patients without being influenced by the Investigators.

The PDQ-39 can also provide an indirect indicator of non-motor symptoms in Parkinson’s disease. Non-motor symptoms have, as a whole, a greater impact on HRQoL than motor symptoms and non-motor symptoms progression contributes importantly to HRQoL decline in patients with PD [32].

The pathogenesis of motor fluctuations suggests that other neurotransmitters, in addition to dopamine, contribute to the appearance of the symptoms, and overactive glutamate activity has been shown to play a key role in the progression and especially motor complications of PD.

Glutamate is essential in the control of movement, due to its actions in neural circuits of the basal ganglia [33].

The results of this post-hoc analysis may be explained by the dual mechanism of action of safinamide, which modulates dopaminergic and glutamatergic pathways, providing a meaningful interpretation of its long-term efficacy.

Some limitations must be considered regarding their generalization. Data of a randomized clinical trial can in fact be limited by the eligibility criteria and the high frequency of medical examinations that do not reflect the routine clinical practice. Further investigation is needed to describe the effectiveness of safinamide in usual care settings and in non-selected PD patients.

### Conclusions

In study 018, patients were experiencing fluctuations and motor complications despite a stable, optimized dopaminergic therapy. This situation reflects the progressive decline in PD, causing growing disability and a considerable negative impact on health-related quality of life for both the patients and their caregivers [34].

The add-on of safinamide 100 mg/day was associated with improvements in motor fluctuations and motor functions without increasing troublesome dyskinesia.

Moreover, safinamide treatment favorably affected QoL and ADL, maintaining the efficacy in the long-term.

Despite some limitations, the findings of this *post hoc* analysis suggest that safinamide may be considered an appropriate adjunct therapy to L-dopa in PD patients not sufficiently controlled. Prospective studies are warranted to investigate the potential benefits of safinamide in comparison with other anti-Parkinson drugs and in real-life situation.

## CONFLICT OF INTEREST

Wolfgang Jost is member of the Scientific Advisory Board of Zambon SpA; Carlo Cattaneo is a Zambon SpA employee; Erminio Bonizzoni is a statistical consultant of Zambon SpA.
